# Use of Proteomics to Explore Biomarkers of Amyotrophic Lateral Sclerosis (ALS): Proof of Principle from Humanized SOD1 Mouse to Human ALS

**DOI:** 10.1021/acsptsci.5c00403

**Published:** 2025-07-28

**Authors:** Nitesh Sanghai, Geoffrey K. Tranmer

**Affiliations:** College of Pharmacy, Rady Faculty of Health Sciences, 8664 University of Manitoba, Winnipeg, Manitoba R3E 0T5, Canada

**Keywords:** amyotrophic lateral sclerosis, proteomics, Western blotting, biomarkers, humanized SOD1 mouse model, target engagement

## Abstract

Amyotrophic lateral sclerosis (ALS) is a rare motor neurodegenerative disease affecting multiple cellular proteins during the progression of the disease. ALS was first discovered by Charcot in 1869, and since then, scientists have been unable to identify a singular cause of the disease. Further, there are no effective treatments available to cure ALS. The benchmark discovery of humanized preclinical SOD1 mouse models, which recapitulates the clinical and pathological phenotypes of human ALS, gives hope to medicinal chemists and neuroscientists around the globe that a suitable drug-like molecule can be discovered and translated into human beings as a means to slow down the progression of the disease. However, little success has been achieved until now in terms of finding an effective treatment for heterogenic and incurable ALS. One area marked for improvement is the use of semiquantitative, antibody-based targeted Western blotting (WB) experiments, which lack the power to analyze multiple cellular events within the entire dysregulated proteomic system. With the inconsistency of WB experiments, unexpected cellular pathways go undiscovered, and hence, loss of translation with no target engagement is seen from preclinical to human clinical ALS. Recent advancements in discovery-based quantitative proteomics have many advantages over WB. These innovative techniques could help solve the inherent problem in WB and their inability to discover multiple altered proteins with the added capability of longitudinal analysis in preclinical SOD1 models, further validating the findings in human ALS. Herein, we applied a holistic approach to summarize various reports on the use of proteomics in ALS from the published literature, and importantly, we found that using a discovery-based proteomics approach in SOD1 preclinical ALS models has revealed a more diverse and global picture of pathological proteins that affect multiple pathways during different stages of disease progression. Furthermore, we found that the proteomic profiling of the humanized SOD1 mouse model provided a proof of principle for translating the diverse pathological biomarker proteins identified in clinical human ALS cases. Moreover, we believe that advancements in the proteomics approach toward ALS biomarkers could bridge the gap between preclinical and clinical studies, enabling scientists worldwide to discover novel biomarkers and treatments that modify the progression of ALS.

## Graphical abstract

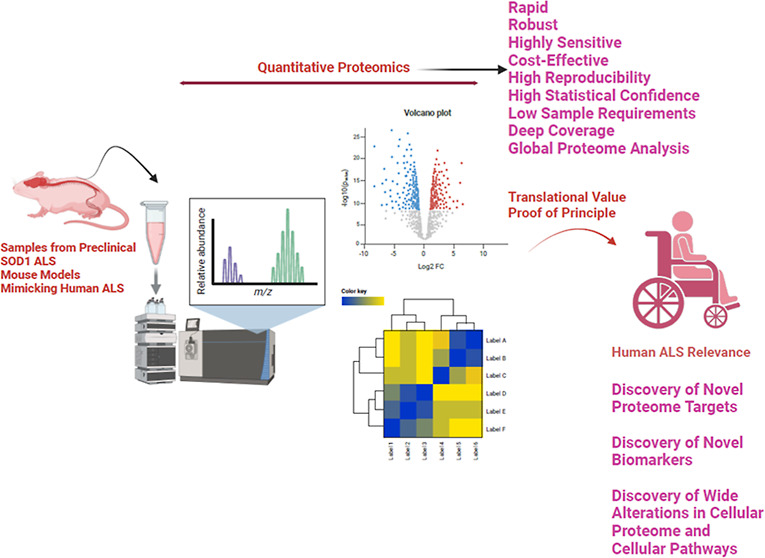

## Brief Insights and Perspectives

- Can quantitative proteomics become the gold standard to measure protein abundance and outperform semiquantitative Western blotting?
- Intriguing proteomic interrogations of humanized SOD1 mouse models of ALS revealed novel dysregulated proteins, which have been validated in human ALS patients.
- Results from humanized SOD1 mouse models, utilizing powerful and robust proteomics tools, provided proof of principle for identifying new biomarkers with translational value in human ALS patients.
- Preclinical humanized SOD1 mouse models are found to be strong predictors of distinct protein-level changes, which could be applied to find novel drugs to slow down the progression of clinical ALS.
- All the reported proteomic profiling was performed in human SOD1mutG93A mice; however, there is a need to profile other mutant SOD1 mouse models.
- Both the familial and sporadic forms of ALS are clinically and pathologically similar. However, to better understand the pathophysiology and heterogeneity in clinical progression of both forms of ALS, it is essential to profile the differential expression of the global proteome longitudinally using preclinical models from both types of ALS.
- In ALS, sex significantly affects disease prevalence, progression, and symptoms. Therefore, utilizing global proteomics to study sex differences is crucial for enhancing healthcare, understanding disease mechanisms, and improving diagnosis and treatment outcomes.
- A proteomics tool could overcome the challenge of biomarker discovery, which could help diagnose and monitor longitudinal disease progression in ALS clinical trials.
- More studies utilizing proteomics tools are warranted to investigate various other preclinical ALS models and unravel the complexity of proteomic changes related to ALS.

ALS is a highly complex and heterogeneous motor neurodegenerative disease resulting from multiple interacting pathophysiological mechanisms. ALS culminates in the disruption of global proteome interacting networks in the brain, spinal cord, and muscle/neuromuscular junctions.^1–3^ To gain insights into the multisystem change in the proteomics of the central nervous system (CNS), highly sensitive, reliable, precise, deep coverage, and reproducible mass-spectrometry-based proteomics approaches have been utilized by the scientific community around the world.^4–6^ Further, proteomic studies have been identified as a reliable tool in detecting and developing novel biomarkers,^7^ which could help in correlating the severity of ALS symptoms and the rate of disease progression.^6,8–11^ Moreover, a global proteomics biomarker approach promotes a better understanding of meaningful drug target mechanisms and achieves positive clinical outcomes.^12–16^

Recently, the scientific community has been debating whether proteomics represents a paradigm shift in the fields of cellular biology and biomedicine. The debate primarily centers on two key questions. First, can proteomics outperform traditional Western blotting (WB) methods? Second, is there a need to cross-validate the results of proteomics with the WB? Explanations have been provided by various experts in the field, based on several convincing rationales.^4,17,18^ First, the use of antibodies, through which scientists determine a specific signal of the protein in a biased and targeted manner, allows for unexpected changes in the cellular components to go unnoticed, and researchers run after the same signal again and again rather than identifying alterations in the signal from the whole cellular system. Hence, WB is a semiquantitative method with a problem of poor specificity, whereas proteomics is a purely quantitative method of analysis (typically several independent peptide fragments of the same protein are targeted to quantify a protein) of whole cellular changes, through which the researchers make sure that the investigated change is the major one out of all changes.^5,19^ Second, with WB, there is a lack of specificity concerning antibodies; several antibodies are absent or may never have existed due to the large number of proteins that have not been studied until now. However, all these proteins with broader coverage can be detected by quantitative mass spectrometry (MS).^4^ Third, in the case of WB, the antibody cannot detect the cellular signal at lower sample numbers (below nanogram levels). In contrast, the latest advancements in MS can detect as low as pico- and femtogram levels with high specificity.^20^ Lastly, researchers use polyclonal antibodies, which provide broader specificity, leading to a high probability of false-positive signals. In contrast, MS analysis offers a large number of peptide identifications with high statistical confidence. Recently, vendors started selling MS-validated antibodies following published guidance from the International Working Group for Antibody Validation (IWGAV).^21,22^ The more significant advantage of exploring distinct cellular events with a single experiment with a lower amount of sample is that it offers an advantage to quantify global proteome changes in the case of rare diseases like ALS, due to the lack of readily detectable and reliable blood and urine biomarkers.

Transgenic mice (Tg) expressing human mutant SOD1 (hmutSOD1) serve as a familial model of human ALS. The expression level of hmutSOD1 is related to the severity and progression of disease in hmutSOD1 mice, as indicated by disease onset, symptom onset, and disease progression. The clinical features or clinical phenotypes in these familial mouse models mimic the progression of familial ALS (fALS) in human disease. The toxic gain of function from these hmutSOD1 proteins causes a change in their biological properties, leading to detrimental misfolding. Further, these toxic aggregates lead to degeneration and, hence, the death of both lower motor neurons in the spinal cord and upper motor neurons in the brain. Some of the most studied and characterized mice for their ALS clinical phenotypes and pathology similar to humans are G93A-SOD1 strain mice^23^ and G37R-SOD1 strain mice.^24^ Both mice are early onset and highly aggressive models that recapitulate human ALS. However, the mechanisms by which these misfolded SOD1 proteins cause toxicity in motor neurons of the CNS are not well understood. These fALS models help to verify the clinical pathogenicity of the novel mutations and also provide clinical insights into the disease mechanisms, crucial for discovering new disease-modifying therapeutics to slow down the progression of ALS in humans.^25^ Since the discovery of SOD1 mutations as a cause of fALS by Rosen et al.^26^ in 1993, there has been no drugs developed that can halt the progression of ALS in SOD1 mouse models and human ALS. Therefore, it is essential to understand the changes in biomolecular proteomic profiling that affect different pathophysiologies associated with ALS. This issue could be solved by identifying different proteins using novel approaches like proteomics. Advances in LC–MS-based global proteome analysis derived from samples of the CNS, including the spinal cord and brain, from different ages of hmutSOD1 and wild-type mice provide a direct assessment of protein differential expression. This novel approach is used to reveal the longitudinal enrichment of protein groups in mice during the progressive stage compared to control mice. To explore different pathological pathways, several emerging reports have utilized proteomics approaches to discover novel proteins associated with neurodegeneration in ALS.

Recently, over the past decade, proteomics has experienced remarkable transformation. MS-based instrumentation and workflows with high speed can identify thousands of proteins affected in both animals and humans. Proteomics is a revolutionary new field in science, used to study the pathological mechanisms of diseases and biomarker-driven drug development. With the unmet need to understand the complex and multifactorial etiology of ALS, proteomics is needed. Proteomics faces challenges such as standardization, validation, and approval as techniques move toward clinical application. Despite this, the field is prepared to transform diagnostics and healthcare, harnessing the plasma proteome’s potential for biomarkers.^27^

However, proteomics has various challenges. First, sample handling, preparation, optimization, and validation. Different biological samples (e.g., blood, CSF, urine, and different tissues) need different sample handling and their optimization for proteomics sampling. Furthermore, quality control for the entire system to perform consistently is required. Second, the challenge of study design, inappropriate study design without considering validated study end points, is a barrier for the success of biomarker drug discovery through proteomics, in the case of ALS, which shows different clinical stages of disease in terms of presymptomatic, disease onset, symptom onset, and death. Furthermore, clinical phenoconversion refers to the transition from a presymptomatic clinical stage with no symptoms to a clinical symptom stage or the onset of the disease. All of these stages should be considered and stratified for proteomic profiling. Each stage of profiling will give insights into biomarker drug development and solve the puzzle of complexity in ALS.^28^ Third, regulatory hurdle is a significant roadblock to clinics. Regulatory hurdles include the strict standardization of processes, biospecimen collection and handling, quality control, instrumentation, clinical validity of proteomics data in patients, data sharing using repositories, and variable costs associated with the proteomics workflow. For example, ALS has different incidence and prevalence rates, with variability in disease presentation across different countries and populations. A combination of genetic, environmental, and lifestyle factors likely influences these differences.^29^ A single proteomics workflow will not be sufficient to solve this problem; instead, different workflows should be developed for each country to identify global proteomics signatures. By addressing these challenges and implementing a standardized, optimized, and validated process across proteomics, the full potential of proteomics can be leveraged in biomarker and drug development^27,30^ Figure 1.

**Figure 1:**
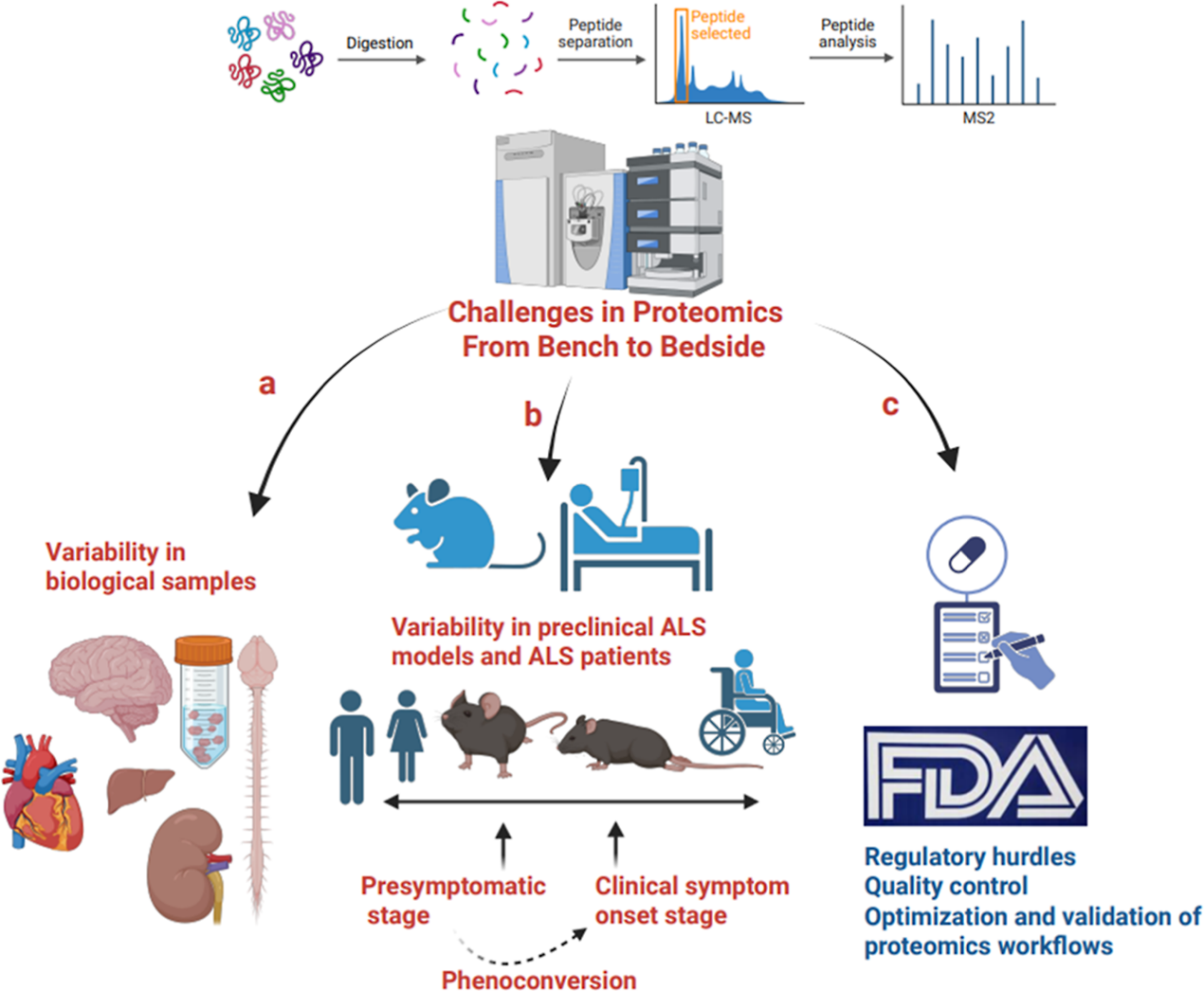
A diagram illustrating the challenges in proteomics profiling from bench to bedside. (a) Variability in sample handling, preparation, optimization, and validation. Different biological samples (e.g., blood, CSF, urine, and other tissues) need different sample handling and their optimization for proteomics sampling. Furthermore, quality control for the entire sample handling system must be performed consistently. (b) Variability in preclinical models and human ALS patients. ALS shows heterogeneity in terms of different clinical stages of disease, including presymptomatic, disease onset, symptom onset, and death. Furthermore, clinical phenoconversion refers to the transition from a presymptomatic clinical stage with no symptoms to a clinical symptom stage or symptom onset of the disease. (c) Regulatory hurdle in terms of strict standardization of process, biospecimen collection and handling, quality control, instrumentations, clinical validity of proteomics data in patients, data sharing using repositories, and variable cost of proteomics workflow.

Herein, we explore the potential of recent advancements in proteomics tools for discovering novel altered cellular proteome pathways in the SOD1 preclinical model of ALS, demonstrating promising translational value in human clinical ALS.

## Discovery-Based Proteomics Approach in Humanized SOD1 Preclinical Models of ALS: Translational Gain from Preclinical to Bedside of ALS Patients

To address the disconnect between the translational shortcomings of preclinical SOD1 mouse models and human clinical ALS, we thoroughly explored recent advancements in proteomics profiling of these models. Our goal is to determine whether proteomics tools can uncover both known and unknown cellular alterations in preclinical models and to assess if these alterations align with pathological findings in human ALS. This supports our belief that SOD1 ALS preclinical models represent an ideal framework for utilizing proteomics to identify biomarkers and develop new drugs aimed at slowing ALS progression.

Lukas and group^31^ investigated the differential expression of proteins in the spinal cord samples of hmutSOD1G93A and wild-type SOD1 Tg mice. They used two-dimensional chromatography coupled with nanospray mass spectrometry with an integrated proteomics–informatics platform to quantify differential protein expression based on the abundance of peptides identified by database searching of mass spectrometry data. During the global proteome analysis, they found that the total extractable protein from hmutSOD1 mice was 30–40% lower compared to those from the wild-type Tg mice and non-Tg mice. The decrease was mainly attributed to the loss of motor neurons (which contribute around 10–15% of proteins) and supporting cells in the hmutSOD1 mice. Further, they found that the mitochondrial proteins are under-represented in the hmutSOD1G93A mice compared to the enhanced representation of mitochondrial proteins in the wild-type Tg mice. Furthermore, investigations of the spinal cord’s global proteome revealed decreased ATP production, accompanied by increased metabolic activity. This hypermetabolism with reduced energy production is due to a decrease in the expression of mitochondrial membrane-bound ATP synthase subunit proteins and an elevation in the expression of antioxidant peroxiredoxin 5 (PRDX5), as well as mitochondrial carbohydrate-metabolizing enzymes such as aldolase, glucose-metabolism-related enzymes such as enolase, and phosphoglycerate mutase.

Interestingly, they also found that a Rab guanine nucleotide dissociation inhibitor (RabGDI) was modestly increased in hmutSOD1G93A spinal cord samples at both the presymptomatic age (60 days) and end-paralytic stage (120 days) compared with non-Tg mice. Additionally, synapsin II protein was downregulated in SOD1G93A mouse spinal cord samples at ages 60 and 120 days compared with non-Tg mice. These results were confirmed and validated through WB experiments on spinal cord samples from both SOD1G93A and non-Tg mice. In addition, they investigated the fact that wild-type SOD1 mice contain only the mitochondrial heat shock protein HSP70, whereas G93A-SOD1 mice contain both cytoplasmic HSP70 and mitochondrial HSP70, which is consistent with the published literature on the localization of cytoplasmic HSP70 in G93A-SOD1 mice. The abundant cytosolic demetalated and misfolded mutant SOD1 binds to HSP70, thereby decreasing the antiapoptotic function of HSP70 and ultimately causing the apoptosis of motor neurons. Moreover, bound SOD1 is unable to cross the membranes of organelles such as mitochondria. Hence, the mitochondrial membrane becomes devoid of SOD1, leading to damage to the mitochondria due to oxidative stress.^32^ The differential regulation of the mitochondrial protein profile in the spinal cord was consistent with the alterations in mitochondrial proteins reported in the published literature for hSOD1G93A mouse models and fALS patients with SOD1 mutations.^32^ Moreover, the synapsin II protein, which is essential for the transport of neurotransmitters^33^ like glutamate and Ý-aminobutyric acid,^34^ is found to be low in the spinal cord of presymptomatic and end-stage mice. Similar alterations in the synaptic vesicle proteins were reported in the anterior horn from ALS subjects.^34^ Recent support for the increase in glycolytic enzyme enolase was found to be consistent with the recent study that demonstrated for the first time the elevation of neuronal-specific enolase in the CSF of ALS patients and further highlighted its importance in being a potential biomarker to distinguish ALS from cervical spondylotic myelopathy.^35^

Ultimately, this global proteomic analysis of spinal cord samples from various stages of mouse development identified proteins that are also altered in the presence of mutant SOD1 in familial amyotrophic lateral sclerosis (fALS). Thus, this study was crucial in identifying unique pathways that could be modulated by the drugs and effectively translated from mouse models to human ALS.

Another study by Chen and colleagues^36^ investigated the proteomic alterations in the lumbar spinal cord of SOD1G93A Tg mice and non-Tg mice at the disease onset age using 4D label-free quantitative proteomics (4D-LFQ) with liquid chromatography–tandem mass spectrometry (LC–MS/MS). Out of 189 differentially expressed proteins analyzed in the lumbar spinal cord samples, 166 were found to be upregulated and 23 were found to be down-regulated. Upon performing protein–protein interaction (PPI) network analysis, Serpina3n or SERPINA3, a serine protease inhibitor, was identified as having the highest ratio between mice reaching the onset of disease (S) and the control (C) (S/C) ratio. The upregulation of SERPINA3 was in agreement with the previously published study, which demonstrates the increased expression of SERPINA3 in preclinical models^37^ and human ALS cases.^38,39^ Moreover, studies have shown the potential of SERPINA3 as a biomarker in the neurodegeneration process because of the presence of SERPINA3 in the neurofilament complex,^40^ in the CSF of progressive multiple sclerosis (MS) patients,^41^ and in the pathological process of AD.^42^ Therefore, SERPINA3 could act as a biomarker in ALS clinical studies.

Furthermore, 48 proteins, primarily from the immune and inflammatory systems, were validated by using parallel reaction monitoring (PRM). Subsequently, KEGG enrichment analysis revealed defective glycolipid metabolism in the early stages of mouse development, which is also evident in both preclinical^43^ and clinical studies.^44^ Moreover, cellular senescence, apoptosis, phagosome, and lysosome pathways were also significantly enriched in the enrichment analysis at the disease onset stage, indicating their role in the death of motor neurons. Further, transcription factor p65 and cell adhesion molecule 1 (Vcam1) in the NF-κB signaling pathway were differentially upregulated in the onset stage of the mice. These results are consistent with reports that demonstrate the significant role of the NF-κB signaling pathway in ALS disease pathology.^45^ Furthermore, the decrease in neuronal NF-κB expression could be an important avenue for slowing the progression of ALS in SOD1 preclinical^46–48^ and clinical cases.^49^ Furthermore, spinal cord samples demonstrated an increased expression of the retinoic acid-inducible gene I (RIG-I)-like receptor signaling pathway. RIG-1 proteins are cytosolic pathogen recognition receptors or act as sensors of viral infection, leading to the transcriptional induction of type-I interferons and cytokine production, essential for antiviral host response.^50^ RIG-1 also plays a novel role in recognizing bacterial pathogens. Recently, Johnson and group^51^ have demonstrated that the human microglial cell line and primary human astrocyte cells in the CNS express RIG-1 proteins, and their expression is upregulated following bacterial infection. The role of RIG-1 in ALS is not well understood. However, the discovery of RIG-1 in spinal cord samples of SOD1G93A mice has increased our understanding of new signaling pathways in preclinical ALS, which could be a potential defective pathway in clinical ALS cases. Furthermore, it could act as a possible target during bacterial infections of the CNS in ALS cases.

Further studies by Perluigi and co-workers^52^ investigated 4-hydroxy-2-nonenal (4HNE)-modified proteins in spinal cord samples using high-copy-number G93A-SOD1 Tg mice. The mice with B6/SJL background survived for around 130 days.^53^ They extracted spinal cord samples from the mice at 120 days of age to examine 4HNE-modified protein modifications during the progression of the disease. The CNS, including the brain and spinal cords, has postmitotic neurons and has high oxygen demands of around 20% of total oxygen. It also has a large pool of transition metal ions like iron and copper and is highly enriched in polyunsaturated fatty acids; therefore, the brain is vulnerable to changes in cellular redox changes and in case of any redox dyshomeostasis causes lipid peroxidation with the end-product 4HNE. Several studies in the past three decades have shown the presence of 4HNE, a toxic chemical in the CNS of both preclinical ALS^54–56^ and human ALS patients.^54,57^ 4HNE is a highly reactive aldehyde and reacts with protein carbonyls via Michael addition to form covalent adducts with amino acids of biomolecules, such as cysteine, lysine, and histidine. These covalent conformational modifications of proteins lead to the death of motor neurons. The 4HNE-modified toxic levels of modified protein products are detected well in both fALS preclinical models^58^ of ALS and human clinical cases.^59^ To explore the role of the 4HNE-modified protein as one of the causes of the progression of disease in SOD1G93A mice, they utilized an A Spec 2E MALDI-TOF (matrix-assisted laser desorption ionization-time-of-flight) mass spectrometer operated in the reflectron mode to generate peptide mass fingerprints. The proteomic investigation in the spinal cords of G93A mice compared with non-Tg mice revealed a significant elevation of 4HNE-modified protein products, including dihydropyrimidinase-related protein 2 (DRP-2), heat-shock protein 70 (HSP70), and α-enolase. These three proteins play a vital role in the growth and maintenance of neuronal health. DRP-2 or Collapsin response mediator protein-2 (CRMP2) is a protein that helps in axonal outgrowth or axogenesis, neurite outgrowth, and neuronal differentiation is found to be significantly depleted due to oxidative modification in neurodegenerative diseases like AD and aging.^60,61^ Therefore, these novel findings of an abnormal DRP-2 protein in the spinal cord of fALS mice demonstrate the link between axonal degeneration and loss of neuronal plasticity, which is known to be altered in both preclinical and clinical cases. Recently, the accumulation of phosphorylated pThr509-CRMP1 has been reported in human ALS patients, suggesting it is involved in axonal degeneration.^62^

Furthermore, there was a significant increase in the level of α-enolase, a glycolytic enzyme that catalyzes the dehydration of 2-phospho-d-glycerate to form phosphoenolpyruvate. Inactivation of the glycolytic enzyme due to oxidative modification by 4HNE leads to a defect in glycolysis and, hence, a decrease in energy production in the form of ATP. In addition, 4HNE causes impairment in the glucose transporter activity like GLUT-3, a neuronal glucose transport protein.^63,64^ It is well known from mountain of reports that the energy source glucose is decreased with increased dependency on the fatty acid in the CNS leading to reduced generation of ATP from mitochondria and hypermetabolism^65,66^ and, hence, leads to the death of motor neurons both in the case of preclinical SOD1 models^67^ and in the SOD1 human ALS patients.^68^ The evidence from proteomics supports the notion that pathological free radicals are a major pathway in the degeneration of motor neurons and a pivotal event in the progression of ALS. The increased level of 4HNE and the protein carbonyl products from various scientific evidence are supported by the advent of proteomics results in both animal and human ALS cases, further validating the possibility of biomarkers in ALS.

Xu and group^69^ discovered a proteome profile in the lumbar spinal cord from 120 day-old hmutSOD1G93A and WT mice using an LC–MS/MS-based analyzer. A total of 297 proteins were found to be differentially expressed proteins (DEPs). Of these DEPs, 193 proteins were upregulated, while 104 proteins were downregulated. They found that apart from oxidative phosphorylation, enzymes responsible for the fatty acid metabolism pathway were altered and found to be upregulated. However, enzymes associated with fatty acid degradation were downregulated and include voltage-dependent anion channel 1 (VDAC1), VDAC2, VDAC3, acetylcoenzyme A acetyltransferase 2 (ACAT2), and acyl-CoA synthetase long-chain family member 6 (ACSL6). In addition, the proteins associated with the regulation of purine metabolism were significantly dysregulated in the spinal cords of hSOD1G93A symptomatic mice. These proteins include phosphoribosyl pyrophosphate synthetase 2 (PRPS2), ectonucleoside triphosphate diphosphohydrolase 2 (ENTPD2), adenosine monophosphate deaminase 3 (AMPD3), and 5′,3′-nucleotidase, cytosolic (NT5C). Furthermore, they also observed the downregulation of ATP citrate lyase (ACLY), succinyltransferase (DLST), succinate-CoA ligase (SUCLA2), and malate dehydrogenase 2 (MDH2), which established a defective TCA cycle in ALS. In addition to defective global fatty acid metabolism, they unveiled dysregulation in sphingolipid metabolism, purine metabolism, arginine metabolism, proline metabolism, and folate-mediated one-carbon metabolism using a metabolomics approach in the symptomatic stage of mice. The identified dysregulation in proteomic and metabolic profiling is found to be consistent with the progression of disease according to previously published pieces of literature. The fatty acid metabolism was found to be increased in both the preclinical^43,70^ and human ALS patients.^71–73^ Similarly, previous studies have shown increased levels of sphingomyelin, ceramides, and cholesterol esters due to defective sphingolipid metabolism pathways, probably due to excessive oxidative stress in fALS preclinical^74^ and human ALS patients.^75^ Recent studies also unraveled the SPTLC1 variants, resulting in unrestrained sphingoid base synthesis, causing increased sphingolipid synthesis, and could cause childhood ALS.^76^ Further, studies reported the alteration of proline, arginine, and purine metabolism in both hmutSOD1 mouse models and human ALS patients.^77,78^ In addition, recently published literature from Filho’s group^70^ using the lipidomics approach supported the evidence of increased sphingolipid and galactosyl ceramides in the motor cortex of symptomatic SOD1G93A mice compared to WT mice. Moreover, there were decreased expressions of cardiolipin and a 6-fold increased expression in numerous cholesteryl esters linked to polyunsaturated fatty acids (PUFAs) in the spinal cord tissue of symptomatic mice compared to that of WT mice. This increase was further speculated to be altered due to oxidative stress, and the spinal cord was found to be more prone to alteration in the lipid profile.^75,79^

This study identifies different pathological pathways through proteomics and metabolic profiling, aligning with earlier pathways implicated in ALS. Additionally, it complements new findings that may offer fresh insights into our understanding of ALS.

Casanas and colleagues^80^ recently profiled synaptoneurosome (SN) proteins in the spinal cord samples isolated from the high copy hmutSOD1G93A mouse model both from onset (age 87–114 days) and symptomatic stages (age 120–139 days) using isobaric tags for relative and absolute quantitation (iTRAQ) labeling tandem MS analysis. Overall, their analysis discovered for the first time the role of MAP kinase-interacting serine/threonine kinase 1 (MKNK1, also known as MNK1), a major mRNA translating protein.^81^ They found the significant downregulation of the MNK1 protein in the SC SNs in both the onset and symptomatic stages of SOD1 Tg mice compared to the non-Tg mice, explaining the abnormal synaptic translation and perturbations in local synaptic translation, which starts early at the onset age stage of ALS governed by the MNK1 pathway. Moreover, they also found a decrease in the expression of the glutamate ionotropic receptor AMPA type-1 subunit (GRIA1) also known as GLUH1; GLUR1; GLURA; GluA1; HBGR1; MRD67; MRT76; and dystroglycan 1 (DAG1) in the onset age of hmutSOD1G93A mice. These results were validated by Western blotting experiments. These results are in line of agreement with the metadata analysis of nine published literature which reported a 21% decrease of GluR1 from postonset to end-stage 117+ days (*p* <0.05).^82^ Previous studies have shown that AMPA receptors are important for maintaining synaptic plasticity, and mice lacking AMPA and GluR1 receptors were found to have impaired synaptic plasticity.^83^ These novel findings are crucial for understanding the MNK1 pathways that influence synaptic transmission in both preclinical and clinical human ALS disease progression. Additionally, MNK1 may serve as a potential target for preventing motor neuron death by safeguarding the local synaptic activity around neurons.

Poon and his group analyzed the proteome of the spinal cord isolated from the symptomatic stage of a female hmutSOD1G93A mouse model ALS, using two-dimensional (2D) gel electrophoresis with a robust parallel proteomic approach applying matrix-assisted laser desorption ionization-time-of-flight (MALDI-TOF) mass spectrometry methods. They mainly investigated the effect of overexpressing the G93A-SOD1 mutation on protein oxidation and their alterations. Several reports from emerging studies have demonstrated the misfolding of both human wild-type and mutant SOD1 due to abnormal redox oxidative changes in both the preclinical^84–86^ and clinical human ALS cases.^87–90^ However, it is important to explore how this toxic gain of function of human mutant SOD1 causes alteration in other biomolecules of life including proteins. Therefore, using the proteomics approach, they identified proteins with increased carbonyl content due to oxidized hmutSOD1 mice compared to nonTg mice, such as SOD1, translationally controlled tumor protein (TCTP), ubiquitin carboxyl-terminal hydrolase isozyme L1 (UCH-L1), and αB-crystallin. TCTP is a protein that is associated with the early development of neurons and glial differentiation.^91^ It is a critical protein for microtubule stabilization, calcium-binding activities, histamine release, cell cycle regulation, axonal development in the hippocampus,^92^ and synthesis of proteins and acts as an antiapoptotic protein.^91^ Recent studies have shown the role of TCPT in restoring cognitive functions in preclinical AD models by restoring the synaptic protein expression in the hippocampus.^93^ Thus, the cumulative evidence suggests the profound role of TCPT in maintaining neuronal health. The finding of a decrease in the expression of TCPT in G93A SOD1 mice because of the oxidation of TCPT suggests the recognized impairment of calcium-binding affinity of TCPT and cytoprotective activities. Consistent with this evidence, there are increased Ca^2+^ voltage currents and signaling reported in the motor neurons of G93A SOD1 mice.^94,95^ Further, ALS patients also reported having higher levels of calcium in lymphocytes^96^ and in the intracellular environment, causing neurotoxicity.^97^

Further, UCH-L1 protein is found both in the brain and spinal cord and is involved in the degradation of abnormally misfolded proteins, oxidative modification of UCH-L1 changes the conformation of the UCHL1 protein, leading to decreased function of UCH-L1 thereby leading to a decrease in the clearance of proteins and increase in oxidative stress and, hence, neurotoxicity of neurons.^98,99^ This benchmark finding of decreased expression of the proteasome clearance protein due to its oxidative modification by an increased expression of G93A SOD1 mutation is consistent with recent findings of high levels of UCHL1 in serum and CSF of ALS patients with decreased activity during disease progression, thus acting as a candidate for a biomarker in ALS diagnosis.^100,101^ Another finding of oxidative modification of αB-Crystallin (HSPB5) with its decreased activity and expression in the spinal cord samples of SOD1G93A mice compared to nTg mice has opened doors to understanding the neurotoxicity of the mutant SOD1 protein. αB-Crystallin (HSPB5) is a class of small heat shock proteins (HSPs) that act as a chaperone of the proteasome pathway, thereby helping the aggregation-prone protein to remain in its correct folding state and prevent its abnormal misfolding.^102^ Further, αB-Crystallin and HSP27 mitigate SOD1 aggregation in vitro^103^ and further upregulate in the mutant SOD1 models in the early symptomatic stage (120 days) and symptomatic stage (150 days); however, the expression was decreased in the presymptomatic (80 days) and advanced stages of disease (350 days).^104^ This may be the rescue operation of chaperone activity in the early to symptomatic stage; however, due to oxidative modification of αB-Crystallin, its expression is decreased in aged end-stage mice. Oxidative modification of αB-Crystallin has a higher aggregation propensity than the degradation mechanism, imparting neuroprotection to motor neurons and increasing the abnormal aggregation of αB-Crystallin. Consistent with this notion, aggregated inclusions are known to be present in preclinical and human clinical cases^105^ In addition, this dysregulation was studied in the symptomatic stages of mice, exploring the molecular changes in the end stage. However, further studies are warranted to decipher the changes longitudinally in all stages of disease progression.

Massignan et al.^106^ analyzed the proteome of the spinal cord isolated from the presymptomatic stage (9 weeks) of female hmutSOD1G93A mouse model ALS, using a robust two-dimensional gel electrophoresis-based differential proteomic approach with MALDI-TOF mass spectrometry. They found that the subtle changes in the proteome of presymptomatic mice mainly affected the mitochondrial bioenergetic enzymes involved in ATP production. The altered proteins include upregulation of pyruvate dehydrogenase E1, ATPase β chain, Enoyl-CoA hydratase, and electron transfer flavoprotein α subunit compared with WT mice. The upregulation is speculated to be due to the response of cellular enzymes to mitochondrial dysfunction. This is supported by evidence of defective mitochondrial energy production due to free-radical-mediated oxidative damage in the G93A mouse model during disease progression^107,108^ and in clinical human ALS patients.^109,110^ Another protein, Cyclophilin A (CypA), also known as peptidylprolyl *cis*-/*trans*-isomerase A (PPIA), demonstrated an increased fold change compared to the WT mice. CypA is the main target of the immunosuppressive drug cyclosporine A (CsA), and it is a highly expressed cellular protein and acts as a molecular chaperone that protects from oxidative damage.^110,111^ CypA plays a crucial role through post-translational modifications.^112^ Importantly, studies have shown the presence of increased expression of CypA in the motor neuronal cells from the spinal cord of G93A mice during the progression of the disease.^113^ Furthermore, due to the high affinity of CsA for CypA, these results are well supported by earlier studies that have demonstrated the extended lifespan of G93A mice following CypA intervention.^114,115^ In addition, later studies in 2017 have shown that decreasing extracellular CypA reduces the neuroinflammation and extends the survival of G93A mice.^116^ Furthermore, they found alterations in proteins regulating iron metabolism, including increased differential expression of ferritin heavy chain (FH) and decreased expression of protein iron-responsive element binding protein 1 (IRP1). The alteration in the expression of FH and IRP1 indicates an abnormal increase in the iron concentration during neurodegeneration. Earlier studies and later studies have shown an increase in the pool of IRP1, leading to iron dyshomeostasis in preclinical ALS models;^117^ however, IRP1 downregulation was never reported in the clinical human ALS cases. Therefore, further investigations are warranted with this hint of downregulation in preclinical models. Further, earlier^118,119^ and later studies have shown increased FH in preclinical ALS models.^120^ Recent meta-analysis studies in human ALS patients have shown elevated levels of ferritin (FH) in the serum of ALS patients, indicating an increased iron load in the serum of these patients.^121,122^ This accumulating evidence from different studies supports the fact that iron acts as a primary culprit in the neurodegenerative process during the progression of disease, in both preclinical mice and human clinical ALS patients.

Zhou and colleagues^123^ identified a novel proteome in the ventral roots and lumbar spinal cord isolated from different stages, including presymptomatic and symptomatic mice (aged 45, 90, and 120 days) of the hmutSOD1G93A mouse model of ALS and wild-type hSOD1 mice, using a robust label-free quantitative mass spectrometry method. They investigated a total of 1299 proteins in the spinal cord samples, of which the statistical analysis of the raw data demonstrated 14 proteins that were significantly differentially regulated and shown to be altered in the G93A mice compared to controls. Further, they validated the increase in galectin 3 (Gal3) in the spinal cord of G93A mice and the human ALS spinal cord samples. Furthermore, they observed a rise in the level of Gal3 protein from the presymptomatic stage to the symptomatic stages of mice, suggesting the possibility of Gal3 being a potential biomarker specific to spinal cord tissues. Gal3 is an evolutionarily conserved family of galectin-class proteins that recognize β-galactoside-binding sites of oligosaccharides. They are known to interact with various glycoproteins and glycolipids of other CNS cell types, playing an essential role in maintaining both innate and adaptive immunity. Gal3 is known to be upregulated in reactive microglia in several neurodegenerative conditions.^124^ Gal3 was known to be upregulated in muscles due to motor neurodegeneration during disease progression in G93A mice.^125,126^ Later studies in rats showed that Gal3 causes neuroinflammation by activating the ROS/TXNIP/NLRP3 signaling pathway; therefore, attenuating the expression of Gal3 could be important in mitigating the neuroinflammation process in spinal cord injury.^127^ Later studies in human ALS patients have also strongly validated these findings of increased Gal3 expression in human ALS serum and CSF samples.^128^ Furthermore, Gal3 elevation was reported in aging and MS patients.^129^ Accumulating evidence from the literature suggests that Gal3, as examined through a proteomics approach by Zhou and colleagues, is a significant neurodegenerative protein with potential as a biomarker under neurodegenerative conditions. However, further research is necessary to validate these findings across various studies.

Another proteomic investigation to unravel the molecular signatures in the hind limb (gastrocnemius) and the forelimb (triceps) muscles of the female hmutSOD1G93A model of ALS was carried out by Capitanio et al.^126^ They used two-dimensional differential gel electrophoresis with protein identification by MALDI-TOF and electrospray ionization mass spectrometry for proteomic analysis at different stages of progression of disease, starting from 7 weeks (early stage of disease) and 14 weeks (advanced stage of disease) and compared with nontransgenic C57BL/6 female mice. At week 7, in the gastrocnemius muscle of the hind limbs, Semaphorin-3A (Sema-3A) was found to be the same as that of the control, whereas the phosphoinositide 3-kinase (PI3K) and Rho-associated protein kinase 1 (ROCK1) proteins were significantly downregulated compared to the control. By contrast, at week 7, in the forelimb’s triceps, Sema-3A levels were similar to the control, PI3K was significantly upregulated, and ROCK1 protein expression was significantly downregulated. In week 14, in the gastrocnemius hindlimbs, the expressions of Sema-3A, PI3K, and ROCK1 significantly increased. By contrast, at week 14, in the triceps of forelimbs, the levels of Sema-3A were similar, whereas PI3K and ROCK1 remained downregulated considerably. Several recent studies have reported that ROCK1 inhibition causes axonal regeneration and enhances microglial function, thereby increasing the survival of motor neurons.^130^ ROCK is an emerging molecular target for treating neurological conditions, including ALS.^131^ The findings of increased ROCK proteins in G93A mice compared to controls are well supported by later findings of increased ROCK proteins in the tissues of ALS patients^132^ and in the preclinical ALS models. Furthermore, ROCK inhibition enhances the survival of G93A models of ALS.^133^ Recent groundbreaking studies targeting Rho kinase in the G93A model with Fasudil have demonstrated an extension of lifespan and motor neuron survival.^134^ Sema 3 proteins are known to be an axonal repellant protein and increased expression is found to cause axonal degeneration and damaged neuromuscular synapses and plasticity very early in the disease stage,^135^ causing degeneration of motor neurons in the preclinical G93A mouse model^136,137^ and human clinical cases.^138^ Therefore, these subsequent findings from preclinical and clinical cases validated the results from proteomics studies in humanized models of fALS mice. Further, it provides a new target to slow down the progression of the disease. PI3K is a lipid kinase that activates several downstream cascades, activating PI3K–AKT phosphorylation pathways and prolonging neuroinflammation by activating microglial cells.^139^ PI3K is vital for neuronal survival and is a known target to modulate the progression of neurological disease.^140^ Earlier, studies published by Wagey et al. in 1998 ^141^ investigated the increased expression of PI3K in the spinal cords of ALS patients, which correlated with the findings from Capitanio et al. in 2012,^126^ the dysregulation and, hence, the increase in PI3K in muscles of humanized G93A mice in different stages of progression.

Strey and his colleagues performed protein profiling of the spinal cord isolated from different stages, including asymptomatic (6–8 week old), symptomatic paralyzed mice overexpressing mutant SOD1 of hmutSOD1G93A mice, and age-matched wild-type containing hSOD1 mice as controls, using a robust two-dimensional gel electrophoresis and mass spectrometry method. A total of 600 protein spots were identified. However, nine proteins represented around 1.3–1.5% of the total proteins identified and were found to be differentially regulated more than 2-fold in protein abundance (normalized) compared to the control mice. In summary, the protein profiling discovered downregulation of stathmin and increased expression of heat shock proteins (HSPs) 25 and 27, phosphatidylinositol transfer protein-α (PITP-), apolipoprotein E (apoE), peroxiredoxin 6 (PRDX6), and ferritin heavy chain (FHC). These alterations of proteins were observed in paralyzed mice; however, they were absent in symptomatic or WT human SOD1 mice. Stathmin protein is ubiquitous for neuronal development, repair, formation of healthy neuromuscular junctions (NMJs), axonal stability, and the development of motor neurons in the CNS.^142^ Stathmin regulates microtubule dynamics and assembly by acting as a microtubule-destabilizing protein that is essential for cellular function. Phosphorylation of stathmin, a post-translational event, is an important event that regulates the microtubule-destabilizing property. However, increased phosphorylation leads to the loss of tubulin-destabilizing activity, leading to the death of motor neurons.^143,144^ Further, stathmin protein is also a hallmark of TDP-43 pathology in ALS, and rescuing the level of stathmin protein helps mitigate the pathology caused by TDP-43 proteinopathy in ALS patients.^145,146^ The novel findings of decreased expression of stathmin proteins are further consistent with the later findings of the most downregulated proteins in human ALS-affected spinal cord tissues.^147–149^ Peroxiredoxin 6 is an antioxidant enzyme that protects neurons from the damaging effects of oxidative stress.^150^ The upregulation of PRDX6 levels in the spinal cord demonstrates the compensatory mechanism and increased oxidative stress in SOD1 mouse models. These data are well validated by the recent studies by Yamamuro-Tanabe et al. in 2023,^151^ in the preclinical SOD1 models, which showed an increased PRDX6 level in the lumbar spinal cord of the SOD1 mouse model. In addition, expressions of PRDX6 were found to be increased before disease onset in the G93A mouse model,^152^ which is in contrast with these studies and needs further investigations with longitudinal monitoring of PRDX6 in mouse models. However, the levels of PRDX6 in human clinical cases of ALS are still unknown and require further investigation. HSP25 is an ATP-independent chaperone that helps to prevent protein misfolding and facilitates their refolding.^153^ Therefore, the findings of increased expression of HSP25 in the symptomatic stage, where most of the SOD1 aggregates are found, are not unexpected.^154,155^ These findings of increased expression of HSP25 in the proteome profile are supported by the later findings which demonstrated the increased expression of HSP25 in SOD1 mouse models^104,156^ and in the human samples of ALS;^157^ nevertheless, further investigations are needed to identify the role of HSP25 in ALS disease progression. FHC is a vital antioxidant system that provides defense from Fe-mediated damage of neurons and, therefore, acts as a neuroprotective protein in the CNS.^158^ Interestingly, the findings of increased FHC have been certified by later studies that demonstrated increased expression of FHC in SOD1 preclinical ALS models.^117^ Moreover, recent studies have also shown a significant increase in the levels of FHC in the serum of ALS patients.^121,159^ Furthermore, the study by Zheng et al. showed the importance of measuring FHC in the serum of ALS patients as a reliable biomarker for disease differentiation and progression.^160^ Another finding of increased expression of apoE was consistent with both the preclinical^161^ and clinical studies in human ALS patients.^162^ Therefore, these confirmed proteomics findings from preclinical SOD1 models to human ALS cases validate the importance of proteomics in animal models of ALS. This can be used optimistically to discover novel treatments to modify disease progression as well as biomarkers for disease prognosis and diagnosis.

Koehn et al., in 2025,^163^ recently investigated the mechanistic details of sex differences in SOD1G93A ALS mouse models within the spinal cord, the primary site of disease onset, that could guide the development of disease-modifying interventions in ALS starting early to slow down the disease progression. They performed protein profiling of the spinal cords isolated from the spinal cords of 120 day old male and female littermates from WT and SOD1G93A mutant mice. They used untargeted, quantitative proteomics using tandem mass tag acquisition and TMT-labeling-based proteomics on high-resolution mass spectrometric instrumentation. The data sets revealed a significant portion of the spinal cord proteome with different protein abundance levels in SOD1G93A mice versus WT mice.

A total of 6170 unique proteins were identified. The quantified proteins covered a broad dynamic range with over a 105-fold difference between the highest and lowest protein abundances, with similarity between replicates. Among the 6170 quantified proteins, 362 (6%) displayed differential regulation between the wild-type (WT) and SOD1G93A spinal cord samples in either male or female specimens. In detail, 283 proteins exhibited significantly altered abundances in female SOD1G93A samples relative to those in female WT spinal cords. In comparison, 234 proteins showed significant differences in abundance in male SOD1G93A samples compared with male WT spinal cords. Remarkably, 155 proteins demonstrated significant disparities between SOD1G93A and WT spinal cords in both male and female data sets. Moreover, variations within the spinal cord proteome were predominantly influenced by sex, with 79 proteins exhibiting significant differences exclusively in the male data set and 128 proteins showing significant alterations solely in the female data set. The top 50 proteins had significantly higher abundances in the spinal cord of both male and female SOD1G93A mice compared to WT mice. Neuroinflammation-associated proteins are upregulated in both male and female SOD1G93A spinal cords. This includes Gal3, with the highest significant upregulation (male: 5.3-fold, female: 4.7-fold), followed by SERPINA3; male: 4.4-fold, female: 4.0-fold. SERPINA3 is strongly associated with its expression, which is linked to microglial and astrocytic neuroinflammation markers both in ALS patients and in the mouse models of ALS.^37,39^ Further, increased expression of SERPINA3 was found to be enriched in the neurofilament conglomerates of ALS motoneurons. Therefore, positive immunoreactivity of SERPINA3 neurofilamentous conglomerates in motor neurons could be an early pathological biomarker of ALS progression.^40,164^ Further, a significant upregulation of proteins was related to the SOD1 network, including SOD1 (male: 3.0-fold, female: 4.2-fold) and copper chaperone for superoxide dismutase (CCS); male: 2.4-fold, female: 2.5-fold. CCS is known to accelerate the disease progression in the ALS mouse model, which shows increased mitochondrial pathology, primarily characterized by mitochondrial vacuolation and swelling.^165^ CCS helps to mature SOD1 by inserting copper, but in the context of ALS, the expression of human CCS can paradoxically worsen the disease.^166^ Therefore, further studies are warranted to investigate the role of copper delivery in slowing down the progression of ALS in human ALS patients.^167^ Glutamate receptors GRIA4, GRIN2A, and GRIA3 were among the proteins with the highest fold reduction in both male and female SOD1G93A mice relative to those in WT mice. In addition, female cohorts showed additional significant downregulation of glutamate receptors GRIN1 and GRIA2 compared with the mice. The decreased expression of glutamate receptors in astrocytes of ALS mice^168,169^ is well known. The perturbations in glutamate receptors are known to cause neurodevelopmental disorders.^170–172^ However, investigations in human ALS are elusive. Further, ALDH3B1, an enzyme responsible for the metabolism of aldehydes and conferring protection against OS, was found to be highly downregulated in both the male and female cohorts.^173^ Recent integrative microarray analysis, molecular docking, and structural dynamic studies have revealed ALDH3B1 as one of the top target differentially expressed genes, which could be druggable to slow the progression of ALS.^174^ Nevertheless, further investigations of its role in ALS are warranted. Additionally, a notable downregulation of HIF1AN specific to males was discovered. HIF1AN is recognized as a negative regulator of hypoxia-inducible factor 1-α (HIF1A).^175^ Dysfunction in HIF1A is a recognized factor in motor neurodegeneration in both the ALS mouse model^176,177^ and in ALS patients.^178,179^

Additionally, the study revealed 79 male-specific and 128 female-specific distinctions within the spinal cord proteome of SOD1 ALS mice. Utilizing untargeted and unbiased proteomics analysis for the first time, this study illuminated the global proteomic alterations present in both male and female ALS mice, underscoring the importance of the sex-specific differential regulation of proteins. The findings advocate for incorporating sex-specific differences into the development of disease-modifying therapies aimed at slowing the progression of ALS in both preclinical and clinical settings.

Furthermore, the proteome alterations in preclinical ALS models once again proved that the SOD1 mouse model is an excellent model that mimics clinical features, clinical disease progression, and alterations of the cellular proteins. Moreover, the cumulative findings of proteome profiling from the above studies have given evidence to support our conjecture that preclinical SOD1 models of ALS are an important discovery, which could be used in liaison with proteomics tools to bring treatments and biomarkers for creating hope for the ALS community Figure 2.

**Figure 2:**
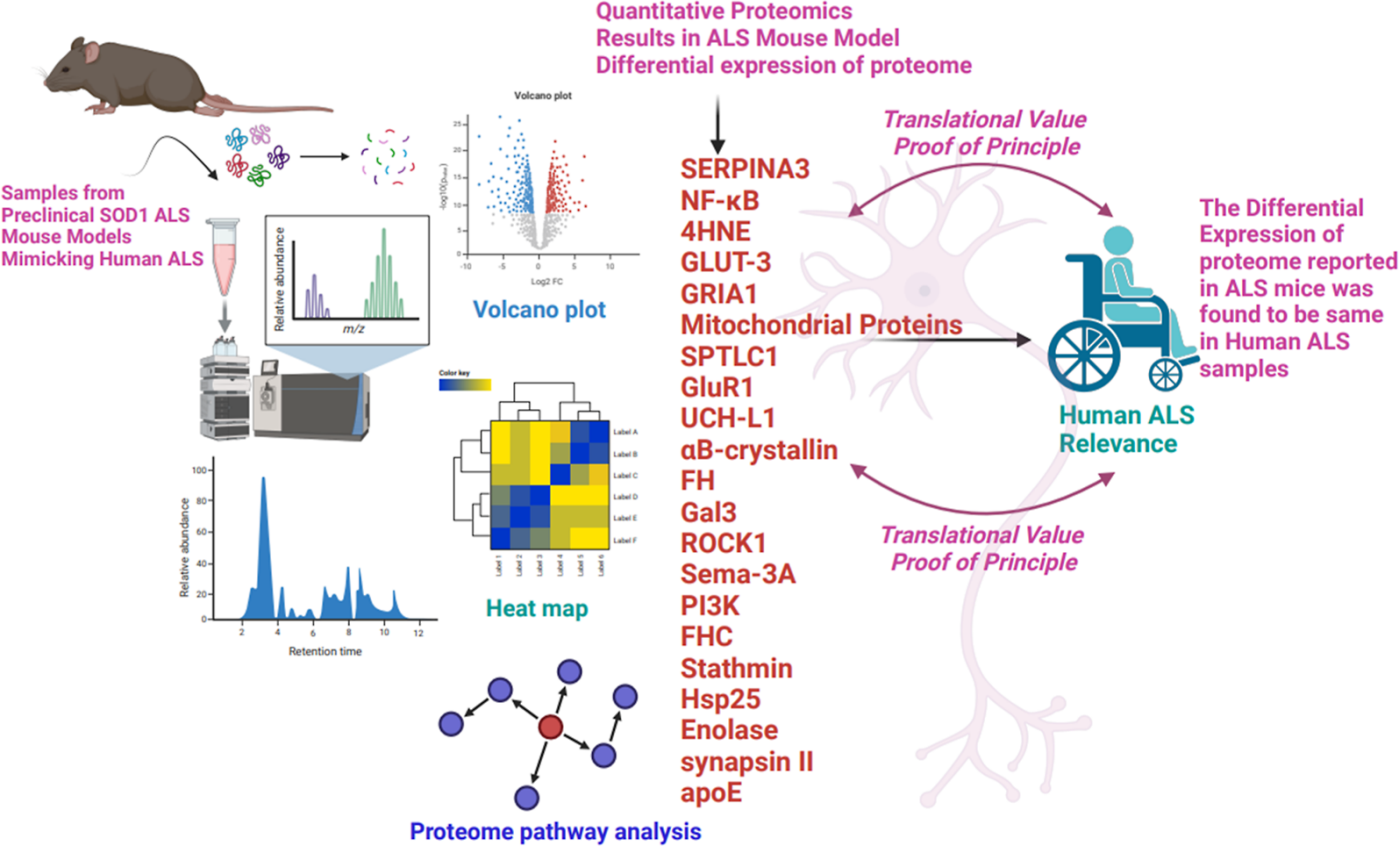
A diagram showing how different alterations in the cellular proteome discovered in preclinical animal models of ALS by using proteomic tools have provided proof of principle consistent with the change of the proteome found in clinical human ALS cases. Proteins were first discovered in SOD1 animal models, and later, the same proteins were reported in human ALS cases.

## Conclusion and Perspectives

ALS is a progressive, rare motor neurodegenerative disease that is always fatal. Furthermore, numerous studies have demonstrated the role of protein misfolding in the pathogenesis of ALS. Thus, we could say that ALS is a neurodegenerative process due to misfolded proteins, which cause toxicity to motor neurons. In addition, these proteins, for example, misfolded SOD1, act like a prion molecule and travel to other parts of the CNS, targeting both neuronal and non-neuronal cells. It is well known that ALS is a multiprotein disease, meaning several proteins are involved in the pathogenesis of neurodegeneration. Therefore, it is essential to employ a tool that could uncover most of the pathological proteins implicated in the pathogenesis of ALS. The drug discovery process requires preclinical data and validation to progress into clinical studies. In a multifactorial disease like ALS, it is not suitable to use conventional semiquantitative and inconsistent WB experiments as this approach is expected to detect targeted signals that can be effectively translated into humans with proof of principle. Further, ALS neurodegenerations have several stages of disease progression, disease onset, symptom onset, and end of life, both preclinical and human ALS. Therefore, it is essential to utilize the latest proteomics tool, which is versatile, robust, and highly sensitive in identifying a diverse range of protein alterations at each stage of the disease, providing insight into different pathological targets and how to modulate them at each stage of progression. Furthermore, we can understand different pathogenic mechanisms through enrichment analysis within a cellular system. In addition to better understanding the effects of treatment of target engagement, a proteomic biomarker is highly needed in a disease like ALS, where the treatments are ineffective in slowing the progression of the disease. We have contemplated this issue of translating findings from preclinical SOD1 models to human clinical ALS. Surprisingly, we found that the proteomics approach used in mouse models is a wise approach to move into clinical studies, because the proteome alterations revealed in SOD1 animal models were later recognized recently in human ALS samples. This validated our notion that using SOD1 mouse models with discovery-based proteomics could be the right approach to translating cellular protein changes in humans. In addition, all the literature published utilizing the proteomics approach has suggested that the preclinical humanized SOD1 mouse models are strong predictors of distinct protein level changes, which could be applied to find novel drugs to slow the progression of clinical ALS. In addition, proteomics should be used in various other preclinical SOD1 models, similar to the early onset, highly aggressive G93A models. One such model characterized by a high expression of SOD1, with early onset and aggressive disease progression, is the G37R mouse model. Utilizing a global proteomics approach could provide us with more in-depth knowledge of the biochemical changes that are disturbed longitudinally in such models, leading to a better understanding. Moreover, the brain and other tissues of interest should be studied by using well-defined time points, with the help of proteomics, to discover the overall picture of pathogenic changes during neurodegeneration.

Furthermore, with this review, we advocate for applying a discovery-based proteomics approach to studying the effects of treatments. This approach will provide an unbiased assessment of treatment effects and has the potential to identify new treatments in a versatile manner, from preclinical to human ALS. We also hope that in the future the use of proteomics-based mass spectrometry will become the gold standard for the more profound discovery of altered cellular pathways in ALS drug discovery.

## Abbreviations

WB: Western blotting
CNS: central nervous system
MS: mass spectrometry
IWGAV: International Working Group for Antibody Validation
hmutSOD1: human mutant SOD1
fALS: familial ALS
PRDX5: peroxiredoxin 5
RabGDI: Rab guanine nucleotide dissociation inhibitor
4D-LFQ: 4D label-free quantitative proteomics
PPI: protein–protein interaction
MS: multiple sclerosis
PRM: parallel reaction monitoring
Vcam1: cell adhesion molecule 1
RIG-I: retinoic acid-inducible gene I
4HNE: 4-hydroxy-2-nonenal
DRP-2: dihydropyrimidinase-related protein 2
HSP70: heat-shock protein 70
CRMP2: collapsin response mediator protein-2
DEPs: differentially expressed proteins
VDAC1: voltage-dependent anion channel 1
ACAT2: acetyl-coenzyme A acetyltransferase 2
ACSL6: acyl-CoA synthetase long-chain family member 6
PRPS2: phosphoribosyl pyrophosphate synthetase 2
AMPD3: adenosine monophosphate deaminase 3
NT5C: 5′,3′-nucleotidase, cytosolic
ACLY: ATP citrate lyase
DLST: succinyltransferase
SUCLA2: succinate-CoA ligase
MDH2: malate dehydrogenase 2
PUFAs: polyunsaturated fatty acids
SNs: synaptoneurosomes
iTRAQ: isobaric tags for relative and absolute quantitation
MNK1: serine/threonine kinase 1
GRIA1: glutamate ionotropic receptor AMPA type-1 subunit
DAG1: dystroglycan 1
MALDI-TOF: matrix-assisted laser desorption ionization-time-of-flight
TCTP: translationally controlled tumor protein
UCH-L1: ubiquitin carboxyl-terminal hydrolase isozyme L1
HSPB5: αB-crystallin
CypA: cyclophilin A
PPIA: peptidylprolyl *cis*-/*trans*-isomerase A
CsA: cyclosporine A
FH: ferritin heavy chain
IRP1: iron-responsive element-binding protein 1
Gal3: galectin 3
PI3K: phosphoinositide 3-kinase
ROCK1: Rho-associated protein kinase 1
Sema-3A: semaphorin-3A
HSP 25,27: heat shock proteins
PITP-: phosphatidylinositol transfer protein-α
apoE: apolipoprotein E
PRDX6: peroxiredoxin 6
FHC: ferritin heavy chain
CCS: copper chaperone for superoxide dismutase
HIF1A: hypoxia-inducible factor 1-α
